# Delayed diagnosis of TAFRO syndrome: A case report

**DOI:** 10.1097/MD.0000000000039148

**Published:** 2024-08-02

**Authors:** Yumeng Qiao, Xin Zhang, Rong Xu, Xiaoyu Jia, Qian Wang

**Affiliations:** a Renal Division, Department of Medicine, Peking University First Hospital, Beijing, China; b Institute of Nephrology, Peking University, Beijing, China; c Key Laboratory of Renal Disease, Ministry of Health of China, Beijing, China; d Key Laboratory of CKD Prevention and Treatment, Ministry of Education of China, Beijing, China; e Department of Hematology, Peking University First Hospital, Beijing, China.

**Keywords:** autoimmune disease, Castleman disease, renal biopsy, TAFRO syndrome, thrombotic microangiopathy

## Abstract

**Rationale:**

TAFRO syndrome is a systemic inflammatory disorder, manifesting as thrombocytopenia (t), anasarca (a), fever (f), reticulin myelofibrosis/renal insufficiency (r), and organomegaly (o), and considered as a unique clinical subtype of idiopathic multicentric Castleman disease (iMCD). Such syndrome gave rise to a clinical picture similar to that of either a connective tissue disease or an autoimmune disease.

**Patient concerns:**

A Chinese young female initially presenting with arthralgia, Raynaud phenomenon, generalized edema, and a positive anti-small nuclear ribonucleoprotein particle antibody was diagnosed as mixed connective tissue disease. The kidney biopsy showed thrombotic microangiopathy. Bone marrow smear showed bone marrow hyperplasia and biopsy revealed suspected light chain restricted expression, megakaryocyte proliferation, and moderate to severe bone marrow fibrosis. A lymph node biopsy was conducted and the histopathological findings were consistent with the subtype of mixed Castleman disease. The clinical symptoms were relieved after regular chemotherapy.

**Diagnoses:**

After above examination results and clinical manifestations, the final diagnoses was TAFRO syndrome.

**Intervention:**

The she was started on chemotherapy with bortezomib, cyclophosphamide, and dexamethasone.

**Outcome:**

After chemotherapy, symptoms such as thrombocytopenia, hematuria and proteinuria disappeared, lymphadenopathy and VEGF level decreased, and bone marrow fibrosis relieved.

**Lessons:**

Our case illustrated the first cases of shared characteristics of mixed connective tissue disease and iMCD-TAFRO syndrome. Cytokines may play a role in the shared pathogenicity of the iMCD-TAFRO syndrome and systemic autoimmune diseases. Therapy directly against inflammatory factors such as corticosteroids or chemotherapy have an important therapeutic implication.

## 1. Introduction

TAFRO syndrome is a systemic inflammatory disorder, manifesting as thrombocytopenia, anasarca, fever, reticulin myelofibrosis, renal insufficiency/reticulin fibrosis, and organomegaly, and considered as a unique clinical subtype of idiopathic multicentric Castleman disease (iMCD).^[1,2]^ Such syndrome gave rise to a clinical picture similar to that of either a connective tissue disease or an autoimmune disease. Here we reported a delayed diagnosis of TAFRO syndrome in a Chinese young female initially presenting with symptoms of arthralgia, Raynaud phenomenon, generalized edema, and a positive anti-small nuclear ribonucleoprotein particle (snRNP) antibody.

## 2. Case presentation

**Figure 1.**
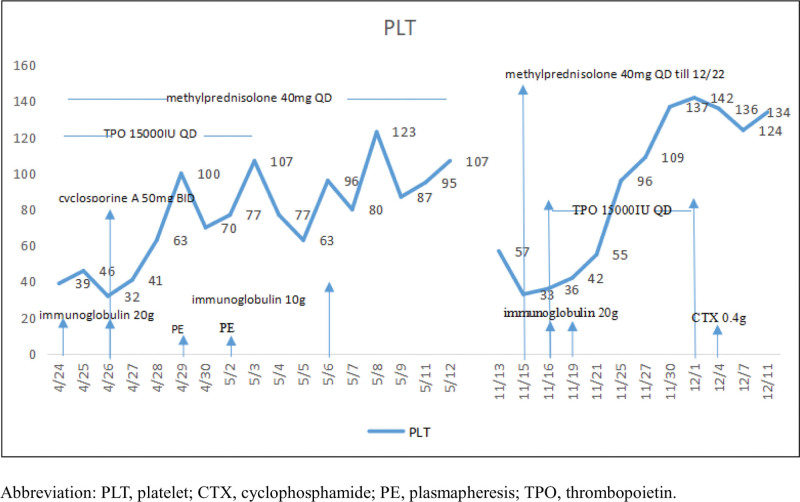
Platelet count change trend chart during treatment from the patient. CTX = cyclophosphamide; PE = plasmapheresis; PLT = platelet; TPO = thrombopoietin.

A 39-year-old Chinese woman who had a 3-year history of arthralgia, Raynaud phenomenon, and generalized edema was admitted to our division because of a recent onset of fever, hypertension, thrombocytopenia, hematuria, and proteinuria. Three years prior to her admission, she was diagnosed as mixed connective tissue disease (MCTD) in a local hospital, presenting with arthralgia, Raynaud phenomenon, superficial lymphadenopathy, and anasarca. The clinical manifestations combined with positive antinuclear antibodies and antibodies targeting the snRNP fulfilled the criteria of MCTD. Steroid therapy (40 mg/day of intravenous methylprednisolone) was initiated for a week and tapered gradually to medroxol 4 mg Qd. Her symptoms resolved until dyeing hair 7 months ago. Due to the above symptoms, the patient went to the local hospital. Immunosuppressive treatment initiated with intravenous methylprednisolone 40 mg QD and cyclosporine A 50 mg BID. Supportive therapy was given simultaneously including antihypertension, plasmapheresis and immunoglobulin infusion due to persistent hypertension, low fever, and purpura. Specific treatment was shown in Figure 1. Her temperature and blood pressure restored to normal but thrombocytopenia and chronic glomerulonephritis persisted. Therefore, she was transferred to our hospital. She had a medical history of hypothyroidism due to Hashimoto thyroiditis and received substitutional treatment with thyroid hormone. She was accidentally diagnosed with lung adenocarcinoma (T1N0M0) of the right inferior pulmonary lobe in 2017 and underwent surgical resection without chemotherapy and radiotherapy.

On admission, her vital signs were normal. A physical examination revealed multiple lymphadenopathies in the bilateral neck, supraclavicular fossa and axilla, and multiple purpuras on the neck and limbs. Laboratory investigations in our hospital revealed the following abnormalities (Table 1): platelet count of 57 * 10^9^/L; microscopic hematuria: 15 to 20/HP; urinary protein: 3+; 24-h urinary protein: 2.42 g/2200 mL, decreased serum complement level: C3 0.588 g/L and C4 level 0.11 g/L; positive antinuclear antibodies 1:1000 (speckled patterns), and positive anti-snRNP antibody 165 IU/mL, but negative anti-dsDNA antibody. She had a normal renal and hepatic function, with normal hemoglobulin, immunoglobulin, CRP, and procalcitonin levels. No monoclonal gammopathy was observed. Ultrasound scan of lymph nodes revealed multiple lymphadenopathies in the bilateral neck, supraclavicular fossa, and axilla, and these enlarged lymph nodes had slightly increased SUV values on PET-CT scan. Combined with these symptoms, signs and laboratory tests, autoimmune diseases especially systemic lupus erythematosus (SLE) were suspected.

**Table 1:** Laboratory findings on admission from the patient.

Parameter	Value	Reference range
*Hematology*		
WBC, 10^9^/L	18.6	3.5–9.5
Hb, g/L	116	115–150
PLT, 10^9^/L	57	125–350
*Blood coagulation*		
PT, seconds	12.30	10.1–12.6
APTT, seconds	27.5	26.9–37.6
D-dimer, μg/mL	0.36	<0.24
*Blood chemistry*		
Alb, g/L	34	40–55
AST, IU/ml	17	13–35
ALT, IU/ml	16	7–40
Scr, μmol/L	67.6	44–133
UA, mg/dL	489	90–360
HCY, μmol/L	19.62	5–14
LDH, IU/L	156	100–240
hs-CRP, mg/dL	1.96	0.00–3.00
*Urinalysis*		
Urine dipstick protein	3+	Negative
Urine occult blood	3+	Negative
24-h urinary protein, g	2.42	< 0.15 g
*Immune index*		
IgG, g/L	24.5	7.23–16.58
IgA, g/L	1.98	0.69–3.82
IgM, g/L	1.46	0.62–2.77
C3, g/L	0.588	0.6–1.5
C4, g/L	0.110	0.12–0.36
C1q, mg/L	151.6	159–233
ANA, IU/mL	1:1000 (speckled patterns)	Negative
dsDNA, IU/mL	negative	Negative
nRNP, IU/mL	153	<25
FH, μg/mL	244.4	247.0–1010.8
IL-6, pg/mL	2.54	<6.4
ADAMTS-13 activity	85%	40% to 130%
VEGF, pg/mL	615.09	0–142.2

ANA = antinuclear antibody; Alb = albumin, ALT = alanine aminotransferase; APTT = activated partial thromboplastin time; AST = aspartate aminotransferase; dsDNA = anti-double-stranded DNA antibody; FH = complement H factor; Hb = hemoglobin, HCY = homocysteine, Hs-CRP = high-sensitivity C-reactive protein; IgA = immunoglobulin A; IgG = immunoglobulin G; IgM = immunoglobulin M, IL-6 = Interleukin-6; LDH = lactate dehydrogenase; PLT = platelet, PT = prothrombin time; Scr = serum creatinine; snRNP = antinuclear ribonucleoprotein antibody; UA = uric acid; WBC = white blood cell; VEGF = vascular endothelial growth factor.

**Figure 2.**
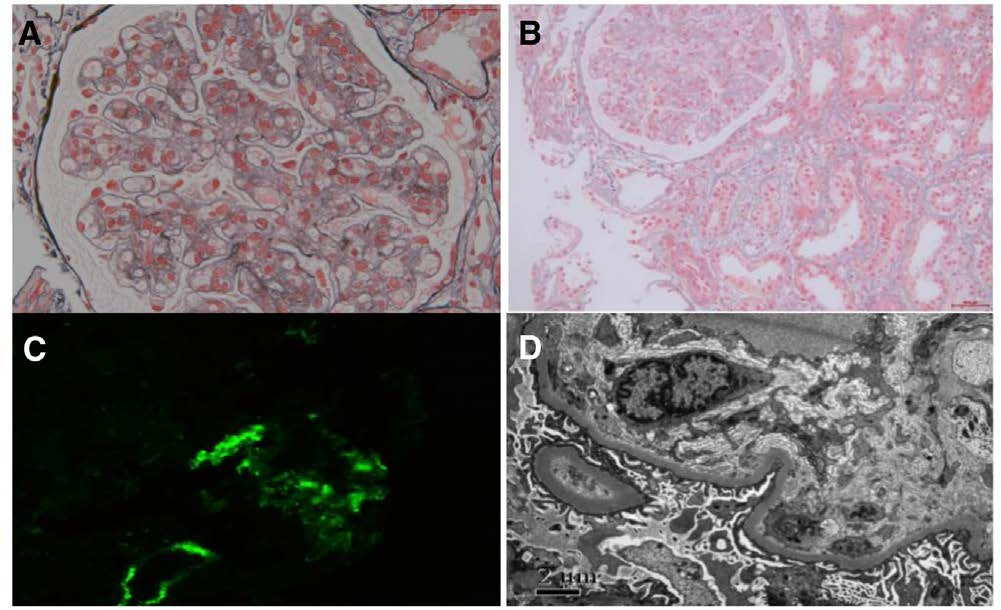
Histopathological findings in renal biopsy from the patient. (A) Diffuse endocapillary proliferation and endothelial swelling (periodic acid-silver metheramine stain [PASM]); (B) fragmented red cells within capillary loops (Masson trichrome); (C) a strong deposition of C3++ along the sclerotic region and segmental mesangial region; (D) diffuse and marked thickening in the inner loose layer of glomerular basement membrane without dense deposit.

Thrombocytopenia and proteinuria relieved after subcutaneous thrombopoietin, intravenous methylprednisolone at 40 mg QD and immunoglobulin treatments (Fig. 1). Kidney biopsy was performed and the pathology was, to our surprise, consistent with thrombotic microangiopathy (TMA)-like lesions (capillary endothelial disease) (Fig. 2). Immunofluorescence showed scarce immunoglobulin deposition (IgA+, IgM+, Ig G‐, C1q‐, FRA‐, Kappa+, Lambda+) but a strong deposition of C3++ along the sclerotic region and segmental mesangial region. Histopathology showed 61 glomeruli, in which diffuse endothelial proliferation and swelling along with obliteration of capillary lumen. Basement membrane thickening with double track sign. Electron microscope showed diffuse and marked thickening in the inner loose layer of glomerular basement membrane with fragmented red cells and without dense deposit.

We further completed TMA-related laboratory workup and revealed normal IL-6 level; elevated vascular endothelial growth factor (VEGF): 615.09 pg/mL (0–142.2 pg/mL); negative cryoglobulinemia; decreased complement factor H (CFH): 244.4 mg/L (247–1010.8 mg/L) and negative CFH antibody. Broken red blood cells were not observed on the peripheral blood smear. ADAMTS-13 activity was within normal range. Bone marrow smear showed bone marrow hyperplasia and biopsy revealed suspected light chain restricted expression, megakaryocyte proliferation, and moderate to severe bone marrow fibrosis. A lymph node biopsy was conducted and the histopathological findings were consistent with the subtype of mixed Castleman disease. The HHV-8 and HIV were negative, so a diagnosis of iMCD was made. Based on her clinical manifestations (thrombocytopenia, anasarca, fever, reticulin myelofibrosis, renal insufficiency, lymphadenopathy) and lymph node biopsy which was consistent with iMCD, we diagnosed the patient with TAFRO syndrome after a multidisciplinary consultation.

Then she was started on chemotherapy with bortezomib, cyclophosphamide, and dexamethasone. Symptoms such as thrombocytopenia, hematuria and proteinuria disappeared, lymphadenopathy and VEGF level decreased, and bone marrow fibrosis relieved after 6 cycles of chemotherapy.

## 3. Discussion

TAFRO syndrome, a rare subtype of iMCD with an estimated annual incidence rate of 0.9–4.9 per million^[3]^ was first reported by Takai et al in 2010.^[4]^ Diagnosing TAFRO syndrome requires all 3 of the following major criteria along with at least one minor criterion, and exclusion of other diseases (Table 2).^[5]^ However, TAFRO symptoms could mimic the features of connective tissue disease, making the diagnosis difficult. Our patient presented with symptoms of arthralgia, Raynaud phenomenon, generalized edema, and a positive anti-snRNP antibody at the disease onset, fulfilled the diagnostic criteria of mixed CTD (High titer anti-RNP antibody accompanied by Raynaud phenomenon and 2 or more of the 3 remaining clinical criteria [swollen fingers, synovitis, and myositis].) and responded well to glucocorticoids therapy at first. After about 3 years of stable condition, the patient gradually presented with skin purpura, fever, hypertension, thrombocytopenia, hypocomplementemia, and nephritic syndrome. Taken together, a diagnosis of SLE was suspected. The re-induction therapy combined with corticosteroids and cyclosporin A achieved a partial remission of thrombocytopenia, but not lasting. The kidney biopsy revealed TMA-like lesions, bone marrow biopsy revealed myelofibrosis, and lymph node biopsy HHV-8 negative Castleman disease. After that, the diagnosis was revised to the iMCD-TAFRO syndrome.

**Table 2:** The diagnostic criteria of TAFRO syndrome.

*The major criteria include*
(1) Anasarca (pleural effusion, ascites, and generalized edema);
(2) Thrombocytopenia (≤100,000/µL);
(3) Systemic inflammation (fever of unknown etiology above 37.5°C and/or serum CRP concentration ≥ 2 mg/dL).
*The minor criteria include*:
(1) Castleman disease-like features on lymph node biopsy
(2) Reticulin myelofibrosis and/or increased number of megakaryocytes in bone marrow
(3) Mild organomegaly (hepatomegaly, splenomegaly, and lymphadenopathy)
(4) Progressive renal insufficiency
*The diseases must be excluded*:
Malignancies; autoimmune disorders; infectious disorders; POEMS syndrome; immunoglobulin G4 (IgG4)-related disease; hepatic cirrhosis; and thrombotic thrombocytopenic purpura (TTP)/hemolytic uremic syndrome (HUS)

The overlapping clinical manifestations between TAFRO syndrome and systemic autoimmune diseases make the differential diagnosis difficult, which may lead to delayed diagnosis. In a review of patients with Castleman disease complicated SLE, Zhang et al found that the percentage of autoimmune thrombocytopenia in such patients was significantly higher than that in the general SLE patients.^[6]^ As lymph node enlargement is common in both active SLE and iMCD, the pathological findings of lymph nodes are crucial for the differential diagnosis. However, some SLE patients had lymphadenopathy shared similar histological features to lymphoproliferative diseases, including Castleman disease.^[7–9]^ Reticulin myelofibrosis, as a minor diagnostic criteria for TAFRO syndrome, was also reported in patients with SLE.^[10]^ These overlapping characteristics between TAFRO syndrome and SLE indicated a shared pathogenesis of both diseases.

So far, IL-6 has attracted the most attention for its role in regulating the acute phase response, T-cell function, and terminal B-cell differentiation. It has been associated with autoimmune disorders, lymphoid malignancies, and Castleman disease.^[11]^ But it was not considered as a major pathogenic cytokine in TAFRO syndrome for the absence of some IL-6 associated symptoms (e.g., thrombocytosis and polyclonal hypergammaglobulinemia). Here in our case, the IL-6 level was normal, implicating cytokines other than IL-6 may play an important role in the pathophysiology of the iMCD-TAFRO syndrome. Iwaki et al recently reported that serum interferon γ-induced protein 10 kDa was elevated in patients with TAFRO-iMCD but not in those with iMCD-NOS.^[12]^ Ginevra et al reported a patient with Castleman disease in 7 years after a connective tissue disorder onset. The plasma concentration of B-lymphocyte stimulator was high in the patient and fell dramatically after chemotherapy, indicating that chronic stimulation of B-cell clones, particularly CD5+, by B-lymphocyte stimulator could favor the development of both autoimmune diseases and a broad range of lymphoproliferative disorders (such as Castleman disease).^[13]^

As kidney dysfunction was reported in more than one-half of TAFRO cases,^[14]^ the kidney histopathological characteristics were rarely reported. A review of recently published case reports mainly revealed MPGN-like lesions or TMA-like glomerulopathy,^[14,15]^ which was consistent with our case. The serum IL-6 and VEGF were considered pathogenic factors. But in our case, besides elevated VEGF, a hypocomplementemia combined with decreased complement factor H level was also noted. Whether the TMA-like lesions were attributed to elevated VEGF or decreased CFH was still unknown. The whole-exome sequencing did not reveal a significant mutation of the complement system, indicating the hypocomplementemia was acquired, possibly after a chronic inflammatory condition caused by systemic autoimmune disorder and TAFRO syndrome.

The optimal treatment for TAFRO syndrome has not been established. The main therapeutic options include corticosteroids, immunosuppressive therapy (e.g., cyclosporin A), rituximab or rituximab-based therapy, and anti-IL-6 therapies.^[14]^ However, these recommendations are essentially based on published case reports and the authors’ own experiences.

To our knowledge, it is the first case reported with shared characteristics of MCTD and iMCD-TAFRO syndrome. As the overlapping symptoms between TAFRO syndrome and the systemic autoimmune disorder usually lead to misdiagnosis, indicating a shared pathogenesis between both diseases. Cytokines other than IL-6 may play a role in the shared pathogenicity of the iMCD-TAFRO syndrome and systemic autoimmune diseases.

## Author contributions

**Conceptualization:** Xin Zhang.

**Data curation:** Yumeng Qiao.

**Formal analysis:** Yumeng Qiao.

**Resources:** Yumeng Qiao.

**Supervision:** Rong Xu, Xiaoyu Jia.

**Writing – original draft:** Yumeng Qiao, Xin Zhang, Rong Xu, Xiaoyu Jia.

**Writing – review & editing:** Qian Wang.
